# Association of Hemoglobin and Myoglobin into Supramolecular Complexes: Significance for Life and Practice

**DOI:** 10.3390/ijms262311700

**Published:** 2025-12-03

**Authors:** Olga V. Kosmachevskaya, Natalia N. Novikova, Alexey F. Topunov

**Affiliations:** 1 Bach Institute of Biochemistry, Research Center of Biotechnology of the Russian Academy of Sciences, 119071 Moscow, Russia; rizobium@yandex.ru; 2 National Research Center “Kurchatov Institute”, 123182 Moscow, Russia; nn-novikova07@yandex.ru

**Keywords:** hemoglobin, myoglobin, hemichromes, amyloids, molecular adaptation of proteins, protein supramolecular complexes

## Abstract

The formation of hemoglobin (Hb) and myoglobin (Mb) supramolecular complexes is examined. These key proteins for oxygen transport and storage undergo conformational transitions, some of which are induced by stress factors, particularly redox-active and toxic substances, e.g., reactive oxygen species (ROS) and reactive carbonyl compounds (RCC). These modifications can lead to partial denaturation, exposure of hydrophobic regions, and loss of stability, promoting self-assembly into high-molecular structures. Reversible associations serve regulatory roles: protein stabilization, transient functional inactivation, and generation of biological signals. Irreversible associations result in the formation of stable aggregates constituting pathological hallmarks of amyloidosis and other proteopathies. Although Hb and Mb fibrillization is not part of their physiological function, under oxidative stress, altered pH, high temperatures, or the presence of post-translational modifications, they can adopt amyloid-like structures characterized by cross-β conformation. Such aggregates exhibit high resistance to proteolysis and accumulate in tissues. Understanding molecular mechanisms behind Hb and Mb aggregation is critical for the diagnosis and timely therapy of amyloid-related diseases. The stability, regular structure, and biocompatibility of Hb and Mb fibrils make them promising for biomedical applications. Functional nanomaterials based on these fibrils are being developed for high-sensitivity biosensors, bioelectronic devices, and nanocarriers for targeted drug delivery.

## 1. Introduction

The formation of protein aggregates is a fundamental biological process that occurs under physiological conditions, such as cytoskeleton assembly, extracellular matrix formation, or induction of liquid–liquid phase separation (LLPS). It also occurs in pathological states such as amyloidosis and other proteopathies [1,2]. The underlying mechanism for most of these processes is the self-assembly of monomers triggered by conformational changes in the protein. These changes enable cells to reorganize their intracellular space in response to stress signals [1,3,4].

Modern cell biology considers the cytoplasm an adaptive compartment capable of rapid phase transitions, during which soluble proteins are transiently assembled into biomolecular complexes or interact with membranes to regulate metabolism, transport biological molecules, and protect against damage [1].

In this regard, hemoglobin (Hb) and myoglobin (Mb)—small globular, α-helical heme-containing proteins serving in oxygen transport and storage—are unique model systems for exploring the general principles of protein self-association. Hb, in particular, has attracted considerable attention as a potent fibrillogenic protein: its exceptionally high concentration in erythrocytes (~5 mM) [5] ensures constant molecular crowding, thereby creating ideal conditions for self-assembly. Depending on the environment, Hb can form either ordered fibrillar aggregates or amorphous complexes. The extensive research into Hb has generated a great deal of data, including the development of accessible bioinformatic tools: HbVar [6], AlphaFold DB [7], and SWISS-MODEL [8]. The presence of a heme moiety with an iron ion (Fe^2+^) in its center renders Hb as an exceptionally useful model system for monitoring conformational dynamics using classical and X-ray spectroscopic techniques [9].

The propensity of Hb and Mb for aggregation, as with other proteins, arises from factors destabilizing their tertiary and quaternary structures—primarily point mutations that disrupt the polypeptide folding and post-translational modifications (PTMs) induced by reactive oxygen species (ROS), reactive nitrogen species (RNS), and reactive carbonyl compounds (RCC). The concentration of these species increases markedly under oxidative and carbonyl stress, which are hallmarks of diabetes, inflammatory diseases, neurodegeneration, and aging [10]. These reactive compounds, e.g., superoxide, peroxynitrite, glyoxal, and methylglyoxal, non-enzymatically modify amino acid residues in proteins, leading to oxidation and glycation. Critically, they can also cause the loss of the heme group—a kind of structural “pin” stabilizing the globular fold (tertiary and quaternary conformations). The long lifespan of erythrocytes (110–120 days) and the cumulative action of stressors on Hb create optimal conditions for the accumulation of modified, destabilized Hb variants that are prone to aggregation.

The interaction of Hb with erythrocyte cell membranes—particularly with band 3 protein and spectrin—is well documented and recognized as a key mechanism underlying hemolysis and erythrocyte cellular signaling [11]. However, our understanding of Hb’s capacity to form supramolecular assemblies in the cell remains severely limited, despite compelling evidence that these processes are involved in the pathogenesis of hemoglobinopathies and erythrocyte senescence.

## 2. Protein Self-Association as a Cellular Response to Stress

### 2.1. Liquid–Liquid Phase Separation in the Cytoplasm

The self-assembly of proteins into aggregates and macromolecular assemblies is now considered not so much a negative process, but rather an adaptive mechanism that enhances the survival of cells under stress conditions. The cytoplasm is now conceptualized as a dynamic, self-organizing system capable of rapidly reconfiguring its physicochemical environment in response to internal and external stimuli [1,3,12].

The cytoplasm is commonly depicted as a viscous “sol”―a homogeneous solution in which metabolic reactions occur. At the same time, it exhibits adaptive rheological properties, reversible transitions between a liquid (sol) state and more ordered, gel-like, or even vitrified states—the so-called “sol–gel transition” [12,13]. In “normal” conditions, the cytoplasm maintains a fluid state that facilitates efficient diffusion and metabolic activity. In contrast, under stress conditions, such as thermal shock, osmotic stress, nutrient deprivation, oxidative stress, acidosis, or DNA damage, the cytoplasm condenses, forming a structured, viscous matrix [14]. This transition serves a protective function: it suppresses metabolic activity to minimal levels, thereby preventing irreversible biomolecular damage and preserving the integrity of cellular components, enabling the cell to enter a metabolic quiescence state. Reversibility is a key advantage of this mechanism: when favorable conditions are restored, the cytoplasm reverts to its liquid state, allowing cells to resume functional activity. This phenomenon exists across a broad phylogenetic range—from bacteria to higher eukaryotes, highlighting the evolutionary conservatism of adaptive strategies at the cytoplasmic level [1,15,16]. Thus, the cytoplasm functions as a self-regulating compartment capable of dynamic homeostasis and protection.

One of the best-characterized mechanisms underlying this reorganization is the liquid–liquid phase separation (LLPS). Under stress conditions, specific proteins and RNAs spontaneously condense into biomolecular condensates—non-vesicular, membraneless compartments. The best-known examples are the so-called “stress granules”, which accumulate non-translating mRNAs, ribosomal subunits, and regulatory proteins, temporarily removing them all from active metabolism. Stress granules form in response to various stresses: heat shock, oxidative and osmotic stress, acidosis, glucose deprivation, and endoplasmic reticulum stress [17]. Through LLPS, the cells reach high spatial organization without expending energy on membrane biogenesis, thus making this process rapid, energy-efficient, and highly controllable. However, when regulation is disrupted by factors such as mutations, aging, or chronic stress, variable liquid condensates can undergo pathological maturation into stable, solid-like aggregates.

Many cytosolic proteins possess an intrinsic ability to self-associate, especially under ATP depletion and proteostatic collapse [18]. Under stress conditions, these proteins assemble into supramolecular aggregations, highly ordered yet reversible complexes that physically isolate sensitive molecules, shielding them from damage. Importantly, these structures do not require energy input to be maintained; they are stable due to weak, multiple interactions, such as π–π stacking, hydrophobic, and electrostatic forces, which are ideal for extreme environments [13,15]. When conditions return to physiological, these aggregations disassemble, and proteins revert to the native functional states [16,19].

Mammalian erythrocytes provide an exceptional model for studying cytoplasmic adaptive mechanisms because they are devoid of organelles, so they cannot synthesize proteins de novo. They are exclusively reliant on post-translational, non-genetic regulatory mechanisms, including reversible protein phase transitions, modification and redistribution of cytoskeletal proteins, interactions with the plasma membrane, and modulation of membrane permeability and mechanical rigidity [20].

### 2.2. Self-Association as a Molecular Adaptation Strategy

**Figure 1:**
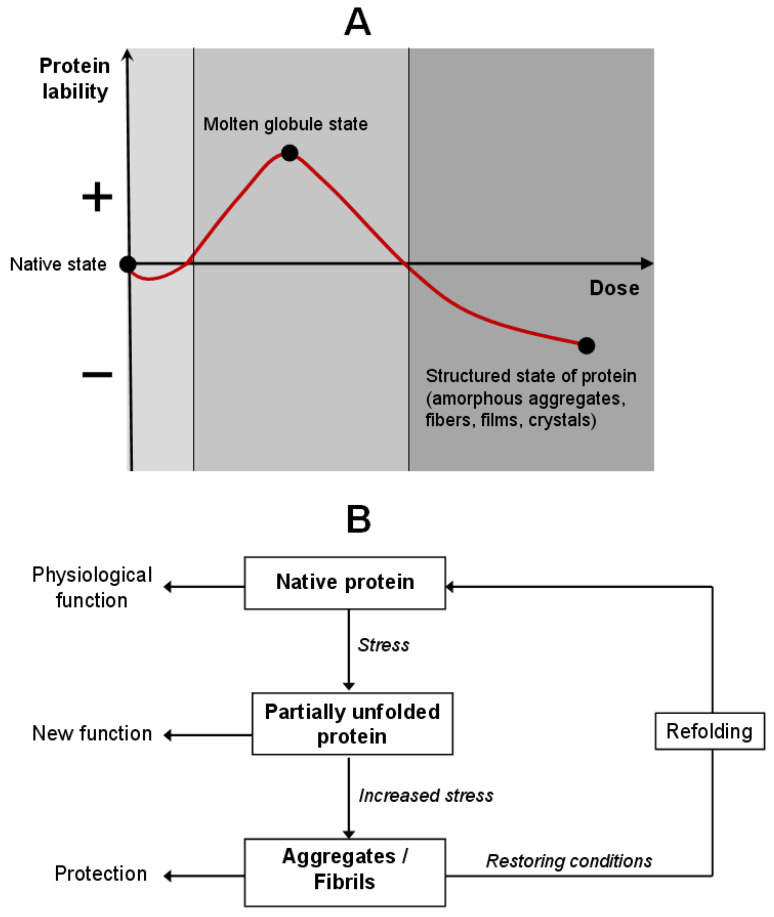
Model of protein adaptation through the hormesis conception. (**A**) Change in protein lability depending on the stressor dose. (**B**) The cycle of protein molecular adaptation: the protein passes through intermediate conformational states, maintaining the native structure or acquiring a new function.

Protein molecular adaptation is a complex, regulated process in which proteins dynamically alter their conformation, stability, and functional properties in response to environmental stressors, often preserving or even expanding their biological activity. Structural changes induced by stress are not merely passive consequences of damage, but an evolutionarily conserved, active survival strategy based on the controlled destabilization and functional reprogramming [21,22]. A model has been proposed wherein protein behavior varies as a function of its lability and stress intensity (Figure 1). This model is interpreted within the framework of hormesis: a mild stress stimulates protective pathways, while a severe one triggers temporary “preservation” mechanisms that safeguard protein structure for recovery upon normalization of circumstances. Hormesis is typically graphically represented by a U-shaped or inverted U-shaped curve, reflecting the ambivalent relationship between the stimulus intensity and biological outcome [23]. This principle is universally conserved from the molecular level to whole organisms, conferring evolutionary advantages through enhanced system plasticity and resistance [24].

Several mechanisms supporting protein adaptation at the molecular level are known. Chaperones (Hsp70, Hsp60, small HSPs) help proteins adopt a correct configuration. The ubiquitin-proteasome and autophagy systems remove the irreversibly damaged proteins. PTMs of proteins (phosphorylation, acetylation, O-GlcNAc modification, ubiquitination, nitrosylation, glycosylation, lipidation, and disulfide bonds) regulate their sensitivity to stress.

Under the physiological conditions, proteins adopt their native state—compact, thermodynamically stable conformation optimally adapted to the environment. Upon mild stress, such as modest temperature elevation, pH shift, moderate oxidative load, or UV irradiation, reversible conformational changes in proteins do not violate their functionality. These alterations can act as allosteric switches, enhancing or suppressing interaction with ligands, substrates, or partner proteins, thereby participating in early stress-response signaling cascades [25].

Under moderate thermal stress (low/high pH, presence of salts, thermal stress, or low concentrations of urea or denaturants), proteins transition into an intermediate conformational state [26,27,28]. This protein state is known as the “molten globule”—a dynamic, partially unfolded yet highly compact structure, characterized by the exposure of otherwise buried hydrophobic domains, cysteine residues, and metal-coordination sites. In this very state, the novel biological functions may be activated, with metal-binding affinity often increasing. Thus, increased conformational lability under such conditions is not pathological but shows molecular plasticity, an adaptive mechanism allowing for structural flexibility without the loss of polypeptide chain integrity.

Under intense or prolonged stress, further destabilization takes place, leading to intermolecular association and formation of aggregates. They may be either functional or non-catalytic but protective, such as amorphous aggregates or vitrified matrices. The latter have been extensively studied in organisms exhibiting desiccation tolerance, including the “resurrection plant” *Craterostigma plantagineum*, certain bacteria, and tardigrades. While drying, the proteins co-assemble with disaccharides and small molecules to form an amorphous, vitrified matrix that physically shields the protein structure from hydrolysis, oxidation, and nonspecific aggregation [29]. Upon rehydration, these proteins rapidly refold and regain full function.

Thus, molecular adaptation of proteins is a multi-layered strategy executed through dynamic reprogramming of structure and function, enabling survival in extreme conditions while preserving the potential for functional recovery.

## 3. Factors of Spontaneous Protein Self-Organization

### 3.1. Amyloidogenic Aggregation of Proteins

Protein self-association is a spontaneous process of molecules assembling into stable oligomeric or polymeric complexes. This mechanism plays a pivotal role in stabilizing protein structures under cellular stress, such as elevated temperature, altered pH, high ionic strength, or oxidative stress. Classic examples of proteins stabilized by self-association include lens crystallins, cytoskeletal filaments (actin, tubulin, intermediate filaments), and viral capsid proteins.

While self-association is often functionally essential, it can also lead to the formation of amyloid aggregates: insoluble, highly ordered structures characterized by a specific β-sheet conformation known as the cross-β structure [30]. In this configuration, β-strands are oriented perpendicularly to the fibril’s long axis, conferring exceptional thermodynamic and proteolytic resistance. The capacity to adopt this structure is not determined by a specific amino acid sequence but rather by fundamental physicochemical properties of the polypeptide chain. It was proposed that almost all polypeptides, under appropriate conditions, can adopt an amyloid fold [31], and the rate and the level of aggregation are modulated by lateral amino acid chains [32].

The process of amyloidogenesis typically proceeds through distinct stages: native monomers undergo conformational rearrangement to form small, highly reactive oligomers; these rapidly convert into protofilaments, which are further organized into mature amyloid fibrils [33,34].

Notably, the most cytotoxic species are not mature fibrils, but soluble oligomers. These intermediates can disrupt the membrane integrity by interacting with transmembrane proteins, perturbing lipid bilayers, inducing ion imbalance, and promoting ROS generation, ultimately leading to cellular dysfunction and apoptosis [35,36].

Despite various primary sequences, amyloid fibrils exhibit remarkable structural conservation. Their shared biochemical and biophysical hallmarks include [37]:

(1) The ability to undergo conformational transition into a β-sheet-rich structure;
(2) Insolubility and resistance to proteolysis, heat, and extreme pH;
(3) Specific binding with diagnostic dyes such as Congo red and thioflavin T.

Almost 40 human proteins have been identified whose amyloidogenic conversion is linked to different diseases, including α-synuclein (Parkinson’s disease), β-amyloid (Alzheimer’s disease), prion protein (Creutzfeldt–Jakob disease), transthyretin (familial amyloidosis), and mutant hemoglobin HbS (sickle cell anemia) [38,39]. Approximately 50 distinct disorders, grouped under the umbrella term “amyloidosis,” are caused by the misfolded proteins [35]. However, amyloid structures are not always pathological. There are many examples of functional (physiological) amyloids with vital specific roles: the storage of peptide hormones (insulin, glucagon, somatostatin) stored in secretory granules of endocrine cells as stable amyloid-like aggregates that prevent their premature activation [40,41]; adhesive matrix in fungi and bacteria (biofilms) [42]; strong fibers in spiders (“spider silk”) [43] and mollusks (byssal threads) [44]; and melanocytic aggregates in skin pigmentation [45].

### 3.2. Mechanisms Initiating Amyloidogenesis

The destabilized native protein conformation, triggered by point mutations or environmental changes (pH, temperature, ionic strength, denaturing agents), initiates aggregation. Meanwhile, the fully denatured proteins tend to form amorphous aggregates that hinder any ordered fibril formation. The key intermediate state that facilitates amyloidogenesis is the molten globule.

In living systems, PTMs profoundly influence the aggregation propensity by altering charge, hydrophobicity, and structural stability. Non-enzymatic glycation—the reaction of amino acid residues (primarily lysine and arginine) with reducing sugars (glucose, fructose) and α-dicarbonyl metabolites (glyoxal, methylglyoxal) is particularly significant. The concentration of these carbonyls increases in diabetes [46,47]. Glycation preferentially affects long-lived proteins like Hb [47,48], generating irreversible cross-links and advanced glycation end-products (AGEs) that stabilize aggregates and impede degradation. Moreover, modification of lysine residues interferes with ubiquitination, the key signal for proteasomal degradation, resulting in an accumulation of damaged proteins [49]. Consequently, AGE-modified proteins are frequently detected in amyloid deposits in patients with diabetic and age-related amyloidosis [34].

Oxidative modifications induced by ROS (superoxide, hydrogen peroxide, peroxynitrite) and redox-active metals (Fe^2+^, Cu^2+^) also disrupt protein structure. These pro-oxidants cause the carbonylation of lysine residues, oxidation of cysteine and tyrosine, loss of cofactors (e.g., heme and metal ions), and global destabilization [50,51]. Oxidative damage synergizes with non-enzymatic glycation: reactive dicarbonyls—products of lipid peroxidation (e.g., malondialdehyde, 4-hydroxynonenal)—are potent glycating agents themselves [52].

Damaged proteins may also acquire novel metal-binding sites. Fe^2+^, Cu^2+,^ and Zn^2+^ ions not only stabilize amyloid fibrils by acting as “molecular staples,” but also catalyze the Fenton reaction, that is, the conversion of H_2_O_2_ into the highly reactive hydroxyl radical (^•^OH), hence amplifying oxidative stress [53,54,55]. Metals can regulate the assembly and disassembly of supramolecular structures, allowing them to be reversible under physiological conditions [56,57].

### 3.3. Aggregation of Hemoglobin and Myoglobin: From Physiological Regulation to Pathological Amyloidogenesis

Hb and Mb proteins are capable of self-associating into amyloid-like fibrillar structures [58,59]. Although the precise mechanisms are not completely understood, the aggregation is governed by a complex interplay of physicochemical factors, PTMs, mutations, and the heme group status.

#### 3.3.1. Factors Promoting Hb and Mb Aggregation

Hb self-association can be triggered by changes in partial oxygen pressure (pO_2_), decreased pH, high protein concentration, prolonged storage, and the presence of mutations. Notably, deoxyhemoglobin (deoxyHb) exhibits a much higher polymerization propensity than oxyhemoglobin (oxyHb).

The first observations of Hb self-association were made in 1970, when the concentrated deoxyHb and oxyHb solutions in concentrated potassium phosphate buffer were found to form gel-like aggregates that dissolved upon dilution, indicating non-covalent interactions between molecules [60]. Electron phase-contrast microscopy showed networks of irregular filaments several nanometers in diameter, occasionally bundling into parallel bunches. Over time, these structures became thick and tortuous before disassembling, demonstrating a sufficiently accelerated aggregation, suggesting a self-propagating nucleation mechanism. Similar self-association also occurs in concentrated Mb solutions [61,62,63].

Hb aggregation occurs not only in vitro, but also in vivo, inside erythrocytes. This process sometimes serves physiological functions, e.g., erythrocyte deoxygenation induces linear Hb oligomerization in chicken, enhancing oxygen-binding cooperativity [64]. In lampreys and diving animals (e.g., the water beetle Anisops), Hb aggregation aids hypoxic adaptation by modulating oxygen affinity and serving as an oxygen reservoir [65].

The most prominent example of pathological Hb aggregation is sickle cell anemia caused by the β6 Glu→Val mutation in hemoglobin S (HbS). Under low pO_2_, deoxyHbS undergoes a conformational change exposing a hydrophobic patch between E- and F-helices, which binds to valine on the neighboring molecule, forming ordered polymers composed of seven pairs of double-stranded chains [38,66,67,68]. These filaments distort erythrocytes, causing vaso-occlusion in the microvasculature.

Other mutant hemoglobins can also aggregate or crystallize, though none exhibit the same fibrillogenicity as HbS (Table 1).

A critical factor enhancing the aggregation potential of both Hb and Mb is the loss of the heme group. Apo-forms (apoHb, apoMb), proteins lacking the prosthetic group, exhibit a dramatically increased propensity to form amyloid-like fibrils [59,69,70]. Heme loss occurs in hemolytic conditions, hemophilia, diabetes, and other pathologies associated with hemolysis and oxidative stress [71].

Studies on mutant apoMb demonstrated that replacing tryptophan-7 and Trp-14 with phenylalanine drastically enhances aggregation, leading to amyloid fibril formation even under physiological conditions [72]. However, adding heme or its structural analogs (niacinamide, chrysin) fully inhibits aggregation and reduces aggregate toxicity, highlighting the protective role of heme in stabilizing the native Mb fold.

PTMs are powerful modulators of aggregation for both Hb and Mb. Acetylation of all 19 lysine residues in apoMb leads to the formation of non-toxic, amorphous aggregates with low β-sheet content and weak binding with thioflavin T [73]. In contrast, native apoMb solutions readily form mature fibrils, indicating that distinct aggregation mechanisms are determined by PTM type. Glycation of Mb by methylglyoxal [74] or glyoxal [75] induces conformational transition from globular to β-sheet-rich structure, yielding both amorphous and fibrillar aggregates starting three days after incubation. Similar effects occur with Hb exposed to glyoxal [75,76]. N-acetylglucosamine glycation also triggers Mb repackaging and amyloidogenesis [77].

Organic solvents such as 2,2,2-trifluoroethanol (TFE) are commonly employed for obtaining Hb fibrils experimentally. At 20% TFE, Hb adopts the molten globule state; at 25%, protofibrils form; and at 45%, mature fibrils are observed [76]. TFE mimics a lipid environment, promoting partial unfolding—a prerequisite for aggregation. Additionally, metallic nanoparticles (e.g., copper) can also induce the molten globule state and promote aggregation of HbA (non-glycated adult Hb) [78,79].

Compelling evidence for the amyloid nature of Hb fibrils was provided by Jayawardena et al. [58], who isolated apoHb from bovine blood and induced aggregation under denaturing conditions. The resulting fibrils were ~5 nm in diameter and up to several micrometers long, exhibiting high thermo- and proteolytic stability (stable from −20 °C to +80 °C, pH 2–10, and resistant to trypsin and organic solvents), along with specific binding to thioflavin T—all hallmark features of authentic amyloids [58].

Fibrils of HbA can also be generated in the presence of 0.5% chloroform, which, according to a proposed model, induces quaternary restructuring and exchange of intersubunit contacts, enabling intermolecular assembly [66].

#### 3.3.2. Intermediate States: Hemichrome and Molten Globule

Hb and Mb aggregation typically initiates with the transition from the native state to a partially unfolded conformation—the molten globule. This state features a fluctuating tertiary structure and exposed hydrophobic surfaces, observed both during in vitro denaturation and folding [80,81]. Studies show that denaturation of Hb and Mb by guanidine hydrochloride proceeds through a reversible intermediate, hemichrome. In hemichrome, the heme iron is in an oxidized, low-spin state coordinated by two histidine residues (bis-histidyl adduct) [82].

**Figure 2:**
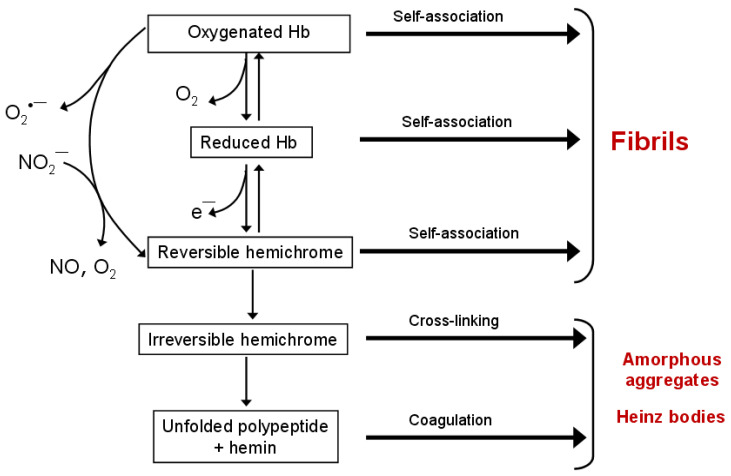
The scheme of Hb denaturation, including intermediate states—hemichromes. Spontaneous or anion-induced autoxidation of oxyHb leads to the formation of oxidized methemoglobin (metHb), reversible and irreversible hemichromes. Hb in all redox and ligand states is capable of forming supramolecular complexes. Reversible hemichromes aggregate into amyloid-like fibrils, while irreversible hemichromes form Heinz bodies.

Hemichromes not only stabilize heme in unstable proteins but also aggregate themselves, interacting with the erythrocyte membrane, particularly with Band 3 protein. This leads to the formation of insoluble inclusions known as Heinz bodies, typical of erythrocytes in hemoglobinopathies caused by Hb with an unstable structure. Structural data indicate a high degree of similarity between hemichrome and the molten globule, suggesting that both represent intermediate states in folding and aggregation pathways [83]. A model for oxyHb denaturation involving autooxidation to metHb, heme dissociation, and subsequent unfolding of apoHb via hemichrome intermediates was proposed [83]. This model explains why unstable mutant hemoglobins accumulate as Heinz bodies and directly links aggregation to the pathophysiology of hemolytic anemias and oxidative stress. Table 2 and Figure 2 summarize the main post-translational oxidative modifications that lead to Hb and Mb aggregation.

## 4. Polymerization of Hemoglobin and Myoglobin via Intermolecular Covalent Cross-Linking

### 4.1. Aggregation via Disulfide Bonds and Metal Ions

Cysteine residues in proteins play essential roles: catalytic, structural, and as redox sensors [84,85]. Cys thiol groups (–SH) in proteins are among the most reactive biological nucleophiles, capable of participating in diverse reversible modifications, including oxidation, nitrosylation, glycation, alkylation, and glutathionylation under mild physiological conditions [11,85]. These reactions play critical regulatory roles in cellular metabolism, particularly under oxidative stress.

The formation of intermolecular disulfide bonds (S–S) between cysteine residues is a common protective response to oxidative damage, e.g., at increased hydrogen peroxide (H_2_O_2_) concentrations. Such covalent cross-links stabilize tertiary and quaternary protein structures, preventing irreversible denaturation. Importantly, disulfide bond formation is often reversible. Enzymes such as thioredoxin and glutaredoxin reduce disulfides back to free thiols at the normalization of cellular redox homeostasis.

Erythrocytic Hb can form high-molecular-weight polymers through intermolecular disulfide linkages, primarily involving Cysβ93. This mechanism has been observed in certain species adapted to hypoxia. For instance, in the freshwater turtle *Trachemys scripta*, Hb polymerizes in response to H_2_O_2_ while retaining full oxygen-binding function [86,87]. These high-molecular-weight Hb polymers are linked by disulfide bonds, protecting cells against oxidative stress. In this context, polymerization acts as a physiological antioxidant mechanism that mitigates oxidative stress within erythrocytes.

A similar disulfide-mediated polymerization is exhibited by human mutant Hb Pôrto Alegre (Table 1), in which an amino acid change exposes an additional cysteine residue, promoting an intermolecular cross-linking [88]. In the house mouse (*Mus musculus*), Hb polymerization occurs via Cysβ13, an amino acid residue missing in human Hb [89]. Human Mb also possesses the unique Cys110 residue, not found in mammalian Mb orthologs, enabling its polymerization under oxidative stress [90].

In addition to disulfide bonding, cysteine residues, along with histidines, are key participants in coordinating transition metal ions (Fe^2+^/^3+^, Zn^2+^, Cu^2+^) [91]. The formation of metal complexes can stabilize misfolded or intermediate protein conformations, alter surface charge distribution, and promote aggregation through intermolecular coordination interactions.

Specifically, Cysβ93 of Hb participates in binding both iron ions [92] and zinc [9]. Previous studies have shown that Zn^2+^ binding induces Hb aggregation [93,94], and atomic force microscopy (AFM) has enabled real-time visualization of Hb coagulation dynamics induced by zinc ions [95].

The capability of metals to induce aggregation is not confined to Hb. Similar phenomena occur in amyloidogenic proteins associated with neurodegenerative diseases, where Zn^2+^, Fe^2+^, and Cu^2+^ promote the formation of toxic oligomers and fibrils [91]. Thus, metal ions can act either as direct cross-linking agents (“metallic staples”) or as catalysts of polymerization, forming stable intermolecular coordination networks.

These properties are actively exploited in biotechnology. Metal-induced polymerization of Hb and Mb enables the design of artificial metalloenzymes, novel biomaterials, and provides deeper mechanistic insights into protein aggregation. Controlled implementation of specific metal-coordination centers allows for the engineering of polymers with assigned properties. For example, the method of metal–phenolic self-assembly was used to produce nanoparticles made of Hb molecules that can bind and release oxygen reversibly [96], a supramolecular Mb polymer was produced in which an external heme cofactor serves as a “molecular hinge,” directing fiber assembly through interprotein interactions. Herewith, Mb preserved the function of the oxygen reservoir.

These approaches have direct medical applications. Polymeric Hb is used to produce Hb-based oxygen carriers (HBOCs)—the potential blood substitutes. Intermolecular cross-linking is essential to reduce colloid-osmotic pressure (tetrameric Hb is rapidly filtered by the kidneys), for extending the half-life in the bloodstream, and for preventing vasoconstriction caused by Hb diffusion into vascular walls. A successful example in question is recombinant polymeric Hb expressed in *Escherichia coli* with site-specific incorporation of surface-exposed cysteine residues. The resulting polymers (~500 kDa MW) exhibited uniform size, no significant increase in arterial pressure, and effective oxygen delivery in vivo [97].

### 4.2. Hb Aggregation via Chemical Cross-Linking Agents

Chemical Hb cross-linking is an intentional strategy aimed at stabilizing the tetrameric structure or generating polymeric complexes. This process requires bifunctional reagents that react with nucleophilic groups, primarily the ε-amines of lysine residues and the α-amines at the N-termini of polypeptide chains. Well-studied cross-linking agents include glutaraldehyde [98], glyoxal (a glycoaldehyde) [99,100], bis(pyridoxal) polyphosphates [101], methylglucopyranoside [102], genipin—a natural plant glucoside [103], polyethylene glycol (PEG) with activated epoxide or carboxyl groups, and peptide-based cross-linkers.

Such modifications aim to decrease Hb’s oxygen affinity to enhance the unloading of oxygen in tissues, increase colloid-osmotic pressure to enhance vascular volume expansion, and prolong circulatory half-life by decreasing glomerular filtration. For instance, glyoxal-crosslinked polyHb in rat models of severe hemorrhagic shock demonstrated efficacy comparable to transfusion of fresh whole blood [100].

A more recent approach harnesses Hb with two β-subunits linked via a PEG spacer [101,102]. Upon polymerization, a supramolecular polymer is generated with an altered Hb–PEG–Hb–PEG architecture, conferring high structural regularity. This method opens new prospects for synthesizing submicrometer-scale polymers with precisely controlled architecture critical for biomaterials and targeted delivery.

Current research focuses on developing the next-generation cross-linked polyhemoglobins that combine high biocompatibility, stability, and minimal immunogenicity. Particular attention is being paid to the use of biodegradable cross-linkers and to site-specific modifications designed to minimize adverse effects, such as vasoconstriction or oxidative stress. While disulfide bonds and metal coordination enable biologically inspired polymerization, chemical cross-linkers provide precise control over polymer size, morphology, and homogeneity. Both strategies are used for developing safe and effective HBOCs, as well as functional biomaterials, including hydrogels, nanoparticles, and supramolecular systems for oxygen storage and delivery.

## 5. Association of Hemoglobin and Myoglobin at the Air–Water Interface

Hb is one of the most extensively studied water-soluble globular proteins with regard to its behavior at the air–water interface (AWI). When we write “water” in this case, we also mean aqueous buffer systems. At the AWI, Hb adsorbs to the interface, forming a monolayer, a thin protein film exhibiting properties of a supramolecular system. The AWI is a convenient model system for investigating protein self-association, as it induces conformational rearrangements that promote aggregation, fibril formation, and gel network assembly [104,105,106,107,108,109].

### 5.1. Protein Adsorption and Conformational Changes

Proteins exhibit surface activity due to their amphiphilic nature: hydrophobic domains minimize contact with water, while polar domains interact favorably with the aqueous phase. Upon adsorption at the AWI, the following processes occur:

- Unfolding (denaturation) of the native structure, exposing buried hydrophobic domains;
- Alteration of secondary structure: reduction in α-helix content and an increase in β-sheet elements;
- Enhanced aggregation due to local concentration effects and a decrease in electrostatic repulsion.

The kinetics of these processes vary significantly across proteins. Albumin and β-lactoglobulin rapidly adsorb (<10 min) with minimal structural perturbation [106]. Lysozyme exhibits slow adsorption kinetics (equilibrium ~2.5 h) but rapid structural changes within 10 min, forming reticular layers enriched in antiparallel β-sheets [106]. Bovine serum albumin loses α-helical content, transitioning into a disordered conformation. Fibrinogen and lysozyme stabilize in β-conformations upon interfacial adsorption [107].

Key drivers of aggregation at the AWI include:

- Concentration effect: Local enrichment of protein at the interface;
- Conformational induction: The AWI stabilizes “aggregation-competent” states [108,109];
- Surface hydrophobicity, which correlates directly with protein damage and aggregation propensity [110,111].

Hydrophobic interactions are central to protein aggregation at the AWI. Studies on the protein FUS (Fused in Sarcoma) demonstrated that a hydrophobic interface at the AWI promotes fibril formation, leading to rigid, cohesive films [111]. Notably, phase transition at the AWI requires ~600 times less protein than for condensation from solution. For bovine insulin, β-lactoglobulin, and chicken egg lysozyme, hydrophobic additives enhance self-assembly into nanofibrils at the AWI [110]. These hydrophobic effects are also critical to the liquid–liquid phase separation (LLPS) in cellular compartments.

### 5.2. Hemoglobin Behavior at AWI

In [112], absorption spectra of oxyHbA and oxyHbS monolayers were analyzed. Both forms exhibited a shift to longer wavelengths in the Soret band compared to their solutions, indicative of protein unfolding and transition toward hemichrome-like states. Surface adsorption isotherms revealed greater unfolding of oxyHbS relative to oxyHbA, consistent with the model proposed by Hirsch et al. [112], which described the denaturation of globular protein at the AWI. More often than not, protein adsorption at AWI is similar to mechanical precipitation at solid–liquid interfaces [113].

The data from studies of the Hb monolayer may help elucidate its structural changes near the erythrocyte membrane. This may be especially important for mutant forms of Hb. Investigations of the kinetics of protein adsorption at AWI revealed distinct behavior of oxyHbS at the interface compared to oxyHbA [113]: The HbS molecule occupies a larger area than the HbA molecule. These differences in surface activity arise from variations in electrostatic, hydrogen bonding, and hydrophobic interactions.

Using the HD-VSFG method (heterodyne-detected vibrational sum frequency generation), changes in the charge of adsorbed Hb at the AWI were studied [114]. It was found that the Hb isoelectric point (pI) in the monolayer was approximately 1 pH unit lower than in solution, indicating significant structural reorganization at the interface. This effect was not observed with other model proteins studied.

X-ray standing wave (XSW) studies demonstrated that Hb in AWI monolayers exhibits an enhanced affinity for binding zinc and iron ions under mild stress conditions (urea, heating, peroxynitrite, hydrogen peroxide) [9]. X-ray absorption near-edge structure (XANES) spectroscopy revealed that each bound Zn^2+^ ion is coordinated by four ligands, two of which are cysteine and histidine residues [9]. Pro-oxidant treatments significantly accelerated metal binding, highlighting the role of the AWI in modulating the metal-binding capacity of heme proteins.

Proteins adsorbed at AWI often undergo partial or complete denaturation upon air exposure, a process known as surface denaturation [115]. In a classic study [116], surface-denatured Hb was estimated to have a molecular weight of ~35 kDa, suggesting dissociation of the tetramer into two dimers. Surface denaturation has been confirmed for bovine Hb adsorbed at its pI point [117]. Glucose oxidase, alcohol dehydrogenase, and urease similarly denature at AWI, forming peptide sheets with a thickness of 8–14 Å [118]. Cross-linking led to the formation of intact enzyme layers at the subphase surface. Cytochrome c at AWI exists as a mixture of native and denatured molecules [119].

Hb monolayers at AWI were used to study protein glycation by glucose [120]. According to adsorption isotherm data, both glucose concentration and incubation time influence Hb’s surface activity. Solution-phase studies indicated that glucose alters local Hb conformation around tryptophan residues and the heme group. Fourier transform infrared (FTIR) spectroscopy of protein monolayers revealed a decrease in the α-helix content and an increase in β-sheet structure, accompanied by aggregate formation [120]. Atomic force microscopy (AFM) showed that in the presence of glucose, Hb film transferred onto a solid substrate transitioned from a globular to an ellipsoidal conformation. The effect of KCl on Hb surface activity and conformation was also examined [121]. KCl increased α-helix content and surface activity, an effect interpretable through the Derjaguin–Landau–Verwey–Overbeek (DLVO) theory of ion–protein interactions. Field-emission scanning electron microscopy (FE-SEM) revealed that Hb films formed in the presence of 0.5 M KCl contained smaller aggregates than those formed without salt.

The influence of pH on human Hb surface activity at AWI was also studied [122], and the Hb monolayer was characterized with LB techniques. Hb in the film existed in tetrameric form. Hb adsorption kinetics depended on both pH and protein concentration in the subphase. Acidic pH promoted the formation of large aggregates, whereas no significant changes occurred at pH 6.8 (Hb’s pI) and alkaline pH. The enhanced aggregation observed under acidic conditions results from α-helix to β-sheet reorganization and from protonation/deprotonation competition at AWI involving aromatic amino acid residues.

Modern synchrotron-based techniques open new avenues for investigating protein monolayers at the air–water interface, enabling the acquisition of highly precise structural information on macromolecules. In the study by Novikova et al. [9], a monolayer of hemoglobin formed at the aqueous subphase in a Langmuir trough was examined using synchrotron-based X-ray absorption spectroscopy (XAS). The results demonstrated that exposure to mild denaturing conditions, specifically 0.09 M urea and moderate heating at 50 °C, significantly enhanced the metal-binding capacity of Hb.

Pretreatment of Hb induced partial destabilization of its tertiary structure, thereby exposing previously hidden metal-binding sites. This structural change enabled Hb to efficiently adsorb Zn^2+^ ions even from highly purified water. Notably, a marked acceleration in metal accumulation was observed during synchrotron X-ray measurements at AWI. We propose that oxidative processes characteristic of the air–water interface may facilitate the activation of metal-binding centers in hemoglobin molecules. An increase in metal content was also detected in intact Hb; however, the rate of metal accumulation was substantially higher in the protein pre-exposed to denaturing factors. These findings highlight the role of the air–water interface in modulating the metal-binding properties of Hb.

Thus, AWI is not merely a physical boundary but a chemically active environment capable of inducing profound conformational and aggregative transformations of proteins. Hb, as a model protein, illustrates how AWI can stabilize pathological conformations (e.g., hemichromes), enhance metal binding, accelerate aggregation at low concentrations, and recapitulate events occurring at biological membranes. These properties of AWI make it a powerful platform for probing the molecular origins of protein misfolding, aggregation, and biomaterial design.

## 6. Functional Nanomaterials Based on Hemoglobin and Myoglobin Fibrils

### 6.1. Applications of Hb/Mb Fibrils in Nanobiotechnology

As noted above, many proteins can self-assemble into amyloid fibrils, thread-like aggregates formed when a protein adopts a non-native, β-sheet-rich conformation. This structural motif confers exceptional resistance to proteolytic degradation, explaining their pathological accumulation in tissues in conditions such as Alzheimer’s, Parkinson’s, and other amyloidoses. At the same time, amyloid fibrils exhibit remarkable thermal, chemical, and mechanical stability: they withstand temperatures up to 284 °C [119], resist organic solvents, extreme pH, and mechanical stress. These properties make them promising materials for various applications under harsh operational conditions, including sterilization and bioengineering systems.

Owing to their stability, high mechanical strength, and amenability to functionalization, amyloid fibrils are increasingly attracting attention as building blocks for biomaterials in biomedicine and biotechnology [123]. They have been successfully employed to fabricate biosensors [123], nanowires [124], nanocomposites [125], thin films [126], nanoporous matrices [127], hydrogels [128], and aerogels [129], as well as substrates for cell culture [130]. Amyloids are also studied as prospects for polymer physics [131], paving the way for the rational design of novel protein-based biodegradable nanopolymeric systems.

For instance, protein-based fibrous nanopolymers provide an attractive alternative to synthetic polymers, traditionally used for the delivery of anticancer drugs and therapeutic proteins [132,133]. Key advantages of protein nanoparticles include high biocompatibility, biodegradability, and low immunogenicity. Nanofibers have been successfully generated from various proteins, including Hb [58,66,71,80]. However, it was only in the study [58] that the complex evaluation of the morphology, structural stability, and functional properties of amyloid fibrils formed from Hb was conducted.

A major limitation to the commercial deployment of amyloid materials is the high cost of purified proteins. In [58], an economically viable approach using bovine blood as a low-cost source of protein was proposed. Fibrils were generated from apoHb under the following conditions: pH 2.8, 125 mM NaCl, incubation at 80 °C for 24 h. The resultant apoHb fibrils demonstrated high stability against industrial solvents used in biosensor and nanowire fabrication. They retained structural integrity after 6 h of trypsin digestion and maintained the initial morphology across a wide pH range.

The study [66] described a molecular mechanism underlying the formation of Hb-based supramolecular structures, forming the basis for designing biocompatible materials. Due to the presence of heme iron, Hb fibrils exhibit electron-transfer capability, endowing them with electrical conductivity and the photovoltaic effect. Upon the illumination at λ = 405 nm, an electrical current was detected on microelectrodes coated with Hb fibrils. Furthermore, HbA fibrils demonstrated a photodynamic effect, suggesting potential applications in phototherapy.

Modern studies focus on developing nano- and micrometer-sized biocomposites based on proteins and lipids [134]. A central challenge is immobilizing proteins on surfaces while preserving their biological activity, since denaturation, unfolding, and aggregation often lead to loss of function [135]. To generate thin protein–lipid films with minimal aggregation, the LB technique at AWI is widely employed [136,137,138]. The LB method is one of the most versatile and controllable techniques for organizing molecules at interfaces, enabling the fabrication of monolayers and multilayers with precise orientation and composition [139,140].

**Figure 3:**
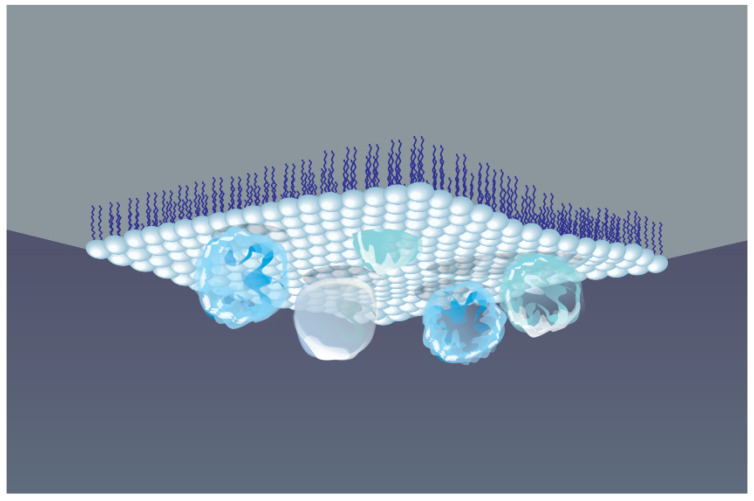
Schematic view of protein macromolecules adsorbed underneath a Langmuir monolayer formed at AWI.

One of the most effective approaches to obtain stable protein monomolecular layers, while maintaining their functional activity, is the adsorption of protein molecules underneath a Langmuir monolayer preliminarily formed at the liquid surface, as schematically shown in Figure 3. These self-assembling processes depend largely on a specific interaction between amphiphilic molecules and protein, which in turn is governed by the formation of covalent bonds or electrostatic effects [136,137,138]. The strength and affinity of these interactions determine the molecular organization of the Langmuir film, its 2D structure, lateral ordering of molecules, as well as conformational rearrangement and thermodynamic stability of adsorbed proteins. Phospholipids, as amphiphiles, are more often used to study the membrane properties of protein–lipid complexes. Whereas polymers are extensively used for numerous industrial applications, e.g., in encapsulation processes, surface modification, pharmaceutical formulations, and the development of optoelectronic devices [141].

For drug storage and controlled release, biomimetic capsules based on protein–lipid combinations mimicking cellular membranes are being developed [142]. One of the best-studied methods is interfacial polymerization. In [142], the formation of highly elastic Hb capsules (0.1–0.3 μm in diameter) within LB films, where the protein retained its secondary structure and thermal stability, was reported. Such elastomeric films can be used to modulate the roughness, porosity, and permeability of polypeptide microcapsules and to encapsulate small peptides and lipophilic compounds.

The LB technique was also used to investigate the self-assembly of Hb with the zwitterionic lipid DPPC (dipalmitoylphosphatidylcholine), a primary component of biological membranes, into hybrid monolayers [134]. The incorporation of Hb into the DPPC layer depends on surface pressure, protein concentration, and interaction time. Within the complex, Hb underwent structural reorganization, with β-sheet domains emerging, while the heme group remained intact and retained its oxygen-binding capacity.

The potential of protein amyloid fibrils in nanobiotechnology is exemplified by the development of next-generation biointerfaces. Hb- and Mb-based nanopolymer structures include encapsulation of the protein within the polymeric matrices to form nanoparticles and other nanoscale architectures.

Hemoglobin and myoglobin are currently used as building blocks for the development of nanomaterials used in various biotechnological tools. These tools operate based on the natural ability of these hemoproteins: binding small gaseous molecules, reversibly altering the redox state of heme iron, and catalyzing the decomposition of hydrogen peroxide to form ROS (Table 3). Hypothetically, amyloid fibrils of Hb and Mb could find application in these biotechnological applications.

### 6.2. ROS-Modulating Activity of Hb and Mb: Application in Biotechnology

Due to the active heme center, Hb and Mb have a number of catalytic properties [154], one of the most studied is the ability to catalyze the decomposition of hydrogen peroxide (H_2_O_2_) by a mechanism similar to that of native peroxidases [155,156,157]:

Hb/Mb (haem-Fe^3+^) + H_2_O_2_ → Compound I (haem-Fe^4+^=O^•+^) + H_2_O (1)

Compound I + electron donor* → Hb/Mb (heme-Fe^3+^) + oxidized product electron donor*: e.g., TMB or ABTS(2)

Thanks to this property, Hb/Mb are used to produce nanozymes—designed nanomaterials with enzyme-mimetic activity for H_2_O_2_ detection (nanozyme-based materials for H_2_O_2_ detection) [146,147] (Table 3). The peroxidase-like activity of Hb/Mb is not used to combat oxidative stress, unlike nanozymes based on native peroxidases, such as horseradish peroxidase [158,159].

The peroxidase catalytic cycle of Hb/Mb is accompanied by the generation of the oxoferryl form (heme-Fe^4+^=O^•+^), which is an extremely strong oxidant that damages proteins, lipids, and nucleic acids. Furthermore, Hb and Mb can generate hydroxyl radicals (^•^OH) via the Fenton reaction:

Fe^2+^ + H_2_O_2_ → Fe^3+^ + ^•^OH + OH^−^ (3)

Thus, Hb and Mb can not only decompose hydrogen peroxide but also enhance oxidative stress [160,161]. This prooxidant effect is being exploited to develop Hb/Mb-based nanocarriers with antibacterial activity [148,149] (Table 3).

To effectively use Hb-based biomaterials as oxygen carriers, it is necessary to minimize the prooxidant effect. To this end, researchers are creating nanomaterials that contain antioxidants. The most common strategy is to functionalize the surface of Hb nanoparticles with antioxidant polymers such as polydopamine and PEG, which scavenge free radicals, protecting the hemoprotein from oxidation to the nonfunctional met form. Another approach is to incorporate antioxidant enzymes (superoxide dismutase and catalase) and/or low-molecular-weight antioxidants (ascorbic acid) into nanostructures [162].

## 7. Conclusions

Intermolecular interactions are one of the most interesting and vital fields of chemistry. Special attention is paid to the formation of molecular assemblies, which is the subject of supramolecular chemistry [163]. Protein–protein interactions are of exceptional interest for scientists working with biological macromolecules, and heme-containing Hb and Mb are not an exception.

These proteins have long been regarded primarily as the specialized oxygen carriers. However, contemporary evidence redefines their role as dynamic proteins endowed with intrinsic fibrillogenic potential encoded in their primary sequence. They can associate into either amorphous aggregates or highly ordered fibrillar structures, depending on various factors: mutations, oxidative stress, heme loss, or proteostasis disruption. Their ability to self-associate may be physiologically beneficial, e.g., for enhancing hypoxia tolerance in diving animals, or pathogenic, underpinning diseases such as sickle cell anemia and hemolytic disorders.

In bioengineering, Hb and Mb are emerging as the next-generation building blocks for advanced biomaterials. Their natural propensity for self-assembly, exceptional thermal stability, biocompatibility, and electronic activity (due to heme groups) make them ideal candidates for generating hybrid hydrogels, oxygen-delivering nanoparticles, bioelectrodes, and even artificial blood substitutes. Precise control over the polymer size, porosity, and functional density can be achieved through intermolecular cross-linking via disulfide bridges, metal coordination, or chemical agents. Moreover, using blood-derived sources, such as donor blood or recycled waste, renders these technologies economically viable and environmentally sustainable. Such materials are already being deployed in the development of artificial lungs, perfusion systems, and implantable sensors sensitive to tissue oxygen levels.

However, the clinical translation of Hb- and Mb-based biomaterials faces a critical challenge: their immunogenicity. Although Hb and Mb are endogenous proteins, their exogenous administration, particularly in modified, aggregated, or heme-deficient forms, can activate damage-associated molecular patterns (DAMPs). Such patterns include free heme and hemin, which bind to Toll-like receptor 4 (TLR4) and CD163, triggering inflammatory cascades via NF-κB and NLRP3 inflammasome activation [164]. It results in macrophage activation, release of pro-inflammatory cytokines (IL-1β, TNF-α), nitric oxide overproduction, and subsequent hypotension and endothelial dysfunction—phenomena previously observed in early clinical trials of cell-free Hb as an oxygen therapeutic. Moreover, even PEGylated or chemically crosslinked derivatives can elicit anti-Hb antibodies upon repeated administration, compromising long-term safety [165]. Notably, Mb, despite its limited plasma circulation under physiological conditions, acts as a potent DAMP upon muscle injury (e.g., trauma or ischemia), capable of inducing systemic inflammation and sepsis-like responses [166]. To overcome these barriers, it is necessary to apply strategies that reduce the immunogenicity of nanomaterials [167].

By applying the innate self-organizing properties of Hb and Mb, we open platforms for innovative biomedical technologies. The successful clinical use of these proteins requires an integrated approach that simultaneously optimizes structural stability, functional integrity, and immunological safety—enabling Hb and Mb full potential to be realized in hypoxia therapy, regenerative medicine, and biosensors.

## Figures and Tables

**Table 1:** Formation of pathological supramolecular complexes by mutant hemoglobins and myoglobins.

Hemoglobin/Myoglobin	Mutation	Type of Supramolecular Structure
HbS	β6 Glu → Val	Forms ordered polymeric filaments resembling amyloid fibrils upon deoxygenation
HbC	β6 Glu → Lys	Prone to intracellular crystallization in homozygous (HbCC) or HbSC compound heterozygotes.
Hb Olympia	β20 Val → Met	Self-assembles into supramolecular forms.
Hb Korle Bu	β73 Asp → Asn	Under oxidative stress (e.g., during infection), it forms intracellular inclusions resembling ordered aggregates.
Hb Zurich	β63 His → Arg	Disruption of heme–protein interaction triggers aggregation into Heinz bodies.
Hb Philly	α35 Tyr → Phe	Mutations at the α1β1-interface induce unusual polymerization or precipitation under specific conditions.
Hb Machida	β6 Glu → Gln	Increased surface hydrophobicity reduces the solubility of oxyHb, promoting aggregation.
Hb Rainier	β145 Tyr → Cys	Oligomerization/aggregation via intermolecular disulfide bonds.
Hb Pôrto Alegre	β9 Ser → Cys	
Mb V68N	Val68 → Asn	Loss of heme → instability → forms amyloid-like fibrils in vitro.
Mb H64Q	His64 → Gln	Reduced O_2_ affinity and protein instability → enhanced aggregation propensity.

**Table 2:** Oxidative modifications of hemoglobin and myoglobin leading to aggregation.

Oxidative Modification	Chemical Changes	Mechanism of Aggregation	Biological Consequences	References
Oxidation of Met, His, Trp	Methionine sulfoxides; oxidized His and Trp derivatives	Conformational changes, protein destabilization	Loss of O_2_ binding; predisposition to degradation and aggregation	[82]
Oxidation of heme iron	Formation of methemoglobin/metmyoglobin	Intermolecular disulfide cross-linking	Aggregation, loss of solubility	[82,83]
Oxidation of cysteine	Disulfide bond formation; sulfinic/sulfonic acid derivatives	Covalent cross-linking of protein molecules	Formation of stable, degradation-resistant aggregates	[26,27,28,50,51]
Oxidation of tyrosine	Tyrosyl radical formation; dityrosine cross-links	Increased hydrophobicity, protein unfolding	Severe oxidative damage; aggregation and proteasome overload	[50,51]
Carbonylation	Addition of carbonyl groups to Lys, Arg, Thr via lipid peroxidation products (MDA, 4-HNE)	Structural disruption, reduced stability	Destabilization of globular fold, increased aggregation propensity	[50,51,52]
Heme destruction	Release of heme and Fe^2+^/Fe^3+^	Hemin induces aggregation; Fe^2+^ enhances oxidative stress via Fenton reaction	Amplification of oxidative damage to proteins and lipids	[53,54,55]
Denaturation and unfolding	Loss of tertiary/secondary structure	Exposure of hydrophobic regions → hydrophobic interactions	Self-assembly into insoluble aggregates	[11,12,13]
Formation of heme–globin complexes	Binding of free heme outside the heme pocket	Conformational changes, self-assembly	Precursors to Heinz body formation	[83]

Abbreviations used in Table 2: 4-HNE (hydroxynonenal), MDA (malondialdehyde).

**Table 3:** Applications of Hb and Mb fibrils in biotechnology.

Protein	Biotechnological Tool	Protein Properties Underlying the Principle of Action	Application Areas	Source
Hb	Oxygen-releasing hydrogels	The natural ability of hemoglobin to bind oxygen at high concentrations and release it at low concentrations, using techniques such as altering the hydrogel microenvironment (e.g., pH or temperature) to control the rate of release.	Wound healing, tissue engineering, treatment of ischemic conditions, therapeutic delivery, and biosensing.	[143,144]
Hb/Mb	Highly sensitive electrochemical biosensors O_2_, CO and NO	The natural ability of heme iron to bind small gaseous molecules (O_2_, CO, NO) with high affinity	Biosensing of small gaseous molecules (O_2_, CO, NO)	[145]
Hb/Mb	Nanozyme-based materials for H_2_O_2_ detection	Hb and Mb intrinsically possess pseudo-peroxidase activity due to their heme iron center.	Highly sensitive and selective biosensors for H_2_O_2_ in various fields: clinical diagnostics, environmental monitoring, and food analysis.	[146,147]
Hb/Mb	Nanocarriers with antibacterial activity	Broad antibacterial activity of the globin part. Heme proteins can enhance peroxidase-like activity, catalyzing the decomposition of H_2_O_2_ to generate ROS.	Destruction of bacteria, including multidrug-resistant strains. Treatment of bacterial septic arthritis, healing of infected wounds.	[148,149]
Mb	Oxygen-sensitive optical biosensor	This approach leverages myoglobin’s natural oxygen-binding function, using changes in its color or fluorescence to indicate oxygen levels.	Monitoring tissue oxygenation, detecting cardiac biomarkers.	[150,151]
Mb	Conductive biointerfaces for nanowires	The ability of the heme group to undergo reversible FeIII/FeII oxidation-reduction reactions makes direct electron transfer between the protein’s redox center and the electrode surface possible.	Highly sensitive biosensors for medical diagnostics, such as for detecting Mb, a biomarker for acute myocardial infarction.	[152]
Mb	Model system for screening aggregation inhibitors	Mb readily forms amyloid fibrils under specific conditions, and its aggregation can be monitored using spectroscopic techniques like circular dichroism and fluorescence.	Study of the influence of different factors (pH, temperature, surfactants) on protein stability and aggregation propensity, and testing of inhibitory compounds.	[153]

## Data Availability

No new data were created or analyzed in this study. Data sharing is not applicable to this article.
